# Childhood Abuse, Social Support, and Long-Term Pharmacological Treatment Outcomes in Patients With Depressive Disorders

**DOI:** 10.3389/fpsyt.2022.803639

**Published:** 2022-02-02

**Authors:** Ju-Yeon Lee, Robert Stewart, Hee-Ju Kang, Ju-Wan Kim, Min Jhon, Sung-Wan Kim, Il-Seon Shin, Jae-Min Kim

**Affiliations:** ^1^Department of Psychiatry, Chonnam National University Medical School, Gwangju, South Korea; ^2^Institute of Psychiatry, Psychology and Neuroscience, King's College London, London, United Kingdom; ^3^South London and Maudsley NHS Foundation Trust, London, United Kingdom

**Keywords:** depression, childhood abuse, social support, pharmacotherapy, treatment outcome

## Abstract

**Objectives:**

This study was performed to investigate the roles of childhood abuse and social support in predicting short- and long-term pharmacological treatment outcomes in outpatients with depressive disorders in a naturalistic 1-year prospective design.

**Methods:**

Patients were recruited at a university hospital in South Korea between March 2012 and April 2017. Subjects with stepwise pharmacotherapy (switching, augmentation, combination, and mixture of these approaches) included 1246 patients at 12-week points in the acute treatment response and 1,015 patients at 12-months in the long-term treatment response. Remission was defined as Hamilton Depression Rating Scale score ≤ 7. Exposure to three types of childhood abuse (physical, emotional, and sexual) before the age of 16 and perceived social support were assessed at baseline.

**Results:**

Individual associations of childhood abuse were associated with poorer treatment outcomes in the 12-month long-term phase, and no significant individual associations were found for social support level with any period outcome. In combination, any child abuse, emotional abuse, and physical abuse were significantly associated with long-term 12-month remission rate in the presence of higher level of social support after adjustment with significant interaction terms. However, no significant interactions were found with sexual abuse.

**Conclusion:**

Synergistic interactive effects of child abuse and social support levels on treatment outcomes in depressive patients were found during long-term pharmacotherapy. Thus, depressed patients with a history of childhood abuse may require specialized clinical approaches, including social support, to enhance the long-term treatment outcomes.

## Introduction

Childhood abuse increases the risk of the development of depression in adulthood (1, 2). Childhood abuse is associated with the severity of adult depression and leads to elevated risk of early onset, recurrent, and chronic depression (3). Various forms of childhood abuse, including emotional, physical, and sexual abuse, are notably prevalent in the general population, with 53.4% of adults in an epidemiological study having experienced at least one form of child abuse in childhood (4). Relative to other forms of child abuse, sexual abuse has received the most empirical attention (5), but there is as yet no consensus on which type(s) of child abuse can specifically predict depression in adulthood.

Childhood abuse has been discussed as an important factor influencing not only the course of depression but also treatment response to pharmacotherapy for adult depression. A meta-analysis of previous studies showed that depressive patients with a history of childhood abuse showed a poorer outcome of pharmacological treatment compared to those without a history of child abuse (odds ratio = 1.26, 95% CI = 1.01–1.56) (6). However, the treatment periods of related studies included in this meta-analysis varied from weeks to several months. Especially, studies with short-term (12-week) treatment revealed that childhood abuse was not associated with response to antidepressants alone (7, 8). Other than the study period, there were also differences in consideration of potential covariates that could contribute to the association between childhood abuse and treatment outcome (9, 10). A study on the interaction between childhood abuse and time over a 12-week course of pharmacotherapy indicated that probability of remission increased significantly faster over time for patients without a history of clinically significant abuse compared to those with such a history (9). They suggested that the gap in remission rates according to time may be due to psychosocial issues beyond medication effects. Therefore, there is a need for investigation into psychosocial moderators that could affect treatment outcomes over time in adults with a history of childhood abuse.

Social support, which refers to an individual's sense that they are cared about and held in positive regard by those in their support networks, is an important psychosocial factor related to the associations between a history of childhood abuse and mental health outcomes (10–12). Previous studies showed that social support buffered the impact of child abuse on risk of depression in adulthood (13–15). Perceived social support was suggested to be associated with reduced pathogenic effects of life stress and to be a protective factor against current depression among adults with a history of childhood adversity (16–18). A prospective cohort study reported that social support played a significant role in moderating the relationship between childhood abuse and subsequent depression in adulthood (19). The importance of social support in affecting disease outcomes was suggested to involve physiological as well as psychosocial mechanisms (20). However, to our knowledge, there have been no previous studies regarding the role of social support as a moderator of a history of child abuse on depression treatment outcomes during antidepressant pharmacotherapy.

The present study was performed to examine whether there is a critical period in which childhood abuse as a predictor of poorer outcome affects antidepressant treatment response during the long-term phase, to examine the interactive effects of childhood abuse and social support on the treatment outcome during pharmacotherapy, and to determine whether there are differences in these associations according to type of child abuse. To address these issues, we analyzed the impact of predictors based on a longitudinal study involving both acute (12 weeks) and continuation phases (12 months) of long-term stepwise pharmacotherapy.

## Methods

### Study Outline and Design

This study was carried out as a component of the MAKE Biomarker discovery for Enhancing anTidepressant Treatment Effect and Response (MAKE BETTER) program. Details of the study have been published as a design paper (21) and registered with cris.nih.go.kr (identifier: KCT0001332). To reflect real-world treatment settings, participants were enrolled regardless of depression subtype or physical comorbidity. Treatment interventions were also conducted in a naturalistic fashion in determining the type, dose, and regimen of antidepressant and other medications, considering patient preference as well as clinician decisions, but were guided by pre-planned measurements and time points. After a 3-week antidepressant monotherapy period, the next treatment steps with alternative strategies could be initiated every 3 weeks during the acute treatment phase (3, 6, 9, and 12 weeks), and every 3 months during the continuation treatment phase (6, 9, and 12 months). All data on sociodemographic and clinical characteristics at baseline and treatment-related variables at follow-ups were obtained using a structured clinical report form (CRF) by clinical research coordinators who were blinded to treatment modalities. These staff were trained in CRF implementation and data collection methods by the research psychiatrists. Patients' data were recorded on a CRF, registered on the website of the MAKE BETTER study (http://icreat.nih.go.kr/icreat/webapps/com/hismainweb/jsp/cdc_n2.live) within 3 days, and monitored by data management center personnel. The population in this study is the same as in the previous our study which was carried out as a component of MAKE Biomarker discovery for Enhancing anTidepressant Treatment Effect and Response (MAKE BETTER) program, and the methods might be some overlap with previous study (22). However, in this study, we investigated the roles of childhood abuse and social support in predicting short- and long-term pharmacological treatment outcomes compared to previous study that investigated predictors of relapse in an outpatient clinical sample with depressive disorders receiving stepwise pharmacotherapy based on early clinical decision-making, applying a naturalistic 24-month prospective design.

### Participants

Patients with depressive disorders were consecutively recruited from March 2012 to April 2017 from those who had visited the outpatient psychiatric department of (removed for review) Hospital. All inclusion instances represented new treatment episodes, i.e., taking newly initiated antidepressant treatment, regardless of whether depressive symptoms were first-onset or recurrent. As the primary objective of the MAKE BETTER study was to discover predictive markers for antidepressant treatment response, all participants were enrolled with consent to receive antidepressant-based treatment approaches only. Inclusion criteria were: age > 7 years; diagnosis of major depressive disorder (MDD), dysthymic disorder, or depressive disorder not otherwise specified (NOS), using the Mini-International Neuropsychiatric Interview (MINI) (23), a structured diagnostic psychiatric interview based on the Diagnostic and Statistical Manual of Mental Disorders, Fourth Edition (DSM-IV) criteria (24); Hamilton Depression Rating Scale (HAMD) (25) score ≥14; able to complete questionnaires, understand the objective of the study, and sign the informed consent form. Exclusion criteria were as follows: unstable or uncontrolled medical condition; unable to complete the psychiatric assessment or comply with the medication regimen due to severe physical illness; current or lifetime DSM-IV diagnosis of bipolar disorder, schizophrenia, schizoaffective disorder, schizophreniform disorder, psychotic disorder NOS, or other psychotic disorder; history of organic psychosis, epilepsy, or seizure disorder; history of anticonvulsant treatment; hospitalization for any psychiatric diagnosis other than depressive disorder (e.g., alcohol/drug dependence); electroconvulsive therapy received for the current depressive episode; and pregnant or breastfeeding. All participants reviewed the consent form and provided written informed consent. For participants aged under 16, written consent was obtained from a parent or legal guardian, and written assent was obtained from the participant. All patients gave written informed consent to participate in the study and use their data. The study was approved by the Ethics Commission of the Chonnam National University Hospital Institutional Review Board as it uses de-identified data. It was registered at cris.nih.go.kr (identifier: KCT0001332).

### Main Exposures at Baseline

#### Childhood Abuse

History of childhood abuse was assessed with the Nemesis Childhood Trauma Interview (26). In this semi-structured interview, participants were asked whether they had ever experienced emotional or psychological, physical, or sexual abuse before the age of 16. Emotional abuse was evaluated by asking, “Were you emotionally or psychologically abused, meaning being yelled at, falsely punished, subordinated to your siblings or being blackmailed?”; physical abuse was evaluated by asking, “Were you abused physically, meaning being hit, kicked, beaten up, or other types of physical abuse?”; and sexual abuse was evaluated by asking, “Were you sexually abused, meaning being touched or having to touch someone in a sexual way or pressured into sexual contact against your will?” As these forms of abuse often occur together (27, 28), a broad definition of “childhood abuse” (having at least one type of abuse) was used for the primary analysis. In this study, participants who had experienced more than one of the three types of child abuse were classified as the “present” group. In the present study, the Cronbach's α values was 0.67, indicating reliable internal consistency.

#### Social Support

Social support was evaluated by the Multidimensional Scale of Perceived Social Support (MSPSS) (29). The MSPSS is a brief measure of a respondent's perception of the social support that they receive from three different sources: a significant other, family, and friends, and consists of 12 items rated on a seven-point Likert-type scale (1, very strongly disagree; 7, very strongly agree). The possible score range is between 12 and 84, with higher scores indicating higher degree of perceived social support. As there is no standard cutoff, the total scores of scales were dichotomized at the median into low and high social support groups. Previous study found that the Korean version of the MSPSS was valid and useful for assessing social support in the Korean population (30).

#### Baseline Covariates

The sociodemographic characteristics examined consisted of age, sex, number of years of formal education, marital status (currently married or not), cohabitation status (living alone or not), religion (religious observance or not), occupation (currently employed or not), and monthly income (above or below 2000 USD). The clinical characteristics evaluated were diagnoses of depressive disorders as outlined above with certain specifiers, age at onset and duration of illness(es), number of previous depressive episodes, duration of present episode, family history of depression, and number of concurrent physical disorders (applying a questionnaire enquiring about 15 different systems or disorders).

Assessment scales for investigating symptoms and function were administered. Depressive and anxiety symptoms were evaluated by the Hospital Anxiety Depression Scale-Depression subscale (HADS-D) and anxiety subscale (HADS-A) (31), respectively; quality of life by the EuroQol-5D (EQ-5D) (32); functioning level by the Social and Occupational Functioning Assessment Scale (SOFAS) (24); number of stressful life events by the Life Experiences Survey (LES) (33); subjective perception of stress by the Perceived Stress Scale (PSS) (34); psychological resilience by the Connor-Davidson Resilience Scale (CDRS) (35); and screening for alcohol-related problems by the Alcohol Use Disorders Identification Test (AUDIT) (36). Higher scores on HADS-D, HADS-A, EQ-5D, LES, PSS, and AUDIT and lower scores on SOFAS and CDRS indicate more severe symptomatology. All scales had been formally translated into the Korean language. In addition, their validity and reliability had been confirmed with acceptable levels in Korean setting (37–42).

#### Pharmacotherapy

Details of the treatment in this study have been published previously (21, 43). Prior to commencement of treatment, a comprehensive review was made of the patients' clinical manifestations (e.g., psychotic and anxiety symptoms), severity of illness, physical comorbidities and medication profiles, and history of previous treatments. Minimal and maximal dosages of pharmacological agents were determined considering existing treatment guidelines (44, 45). In the first treatment step, patients received antidepressant treatment, taking into consideration these data and treatment guidelines (45–47), for 3 weeks. The antidepressants used were bupropion, desvenlafaxine, duloxetine, escitalopram, fluoxetine, mirtazapine, paroxetine, sertraline, venlafaxine, and vortioxetine. After the first antidepressant monotherapy treatment step, the next step of pharmacotherapy was administered every 3 weeks during the acute treatment phase (3, 6, 9, and 12 weeks with a 3-day allowable window) and every 3 months during the continuation treatment phase (6, 9, and 12 months with a 7-day allowable window), whenever needed. At the end of each step, overall effectiveness and tolerability were reviewed prior to proceeding with measurement-based next-step treatments. In cases of insufficient improvement (HAMD score reduction <30% from baseline) or intolerable side effects, patients were instructed to choose whether they would prefer to remain in the current step or enter the next step strategy with switching (S), augmentation (A), combination (C), S + A, S + C, A + C, and S + A + C treatment. Patients were also allowed to receive next-step treatment if they showed sufficient improvement (HAMD score reduction ≥30% from baseline) with absence/tolerable side effects. To determine the treatment strategies, each patient's preference was given priority to maximize medication compliance and treatment outcomes (48). The antidepressants switched or combined were bupropion, desvenlafaxine, duloxetine, escitalopram, fluoxetine, mirtazapine, paroxetine, sertraline, venlafaxine, and vortioxetine. Augmented drugs were buspirone, lithium, triiodothyronine, and atypical antipsychotics, including aripiprazole, risperidone, olanzapine, quetiapine, and ziprasidone.

#### Treatment Outcome

Remission was defined as HAMD score ≤ 7. The main outcome measures were remission at 12 weeks and at 12 months.

### Statistical Analysis

Between-group comparisons of baseline sociodemographic, clinical, and treatment-related characteristics according to the presence of any childhood abuse were analyzed using the *t*-test or χ^2^ test, as appropriate. The same comparisons were repeated between groups with lower social support vs. higher social support. Considering these associations and collinearity between the variables, covariates were considered in later adjusted analyses. The individual associations of childhood abuse and social support groups with the 12-week and 12-month remission status were analyzed in logistic regression models before and after adjusting for potential covariates. Interactive effects of childhood abuse and social support on the 12-week and 12-month remission status were analyzed using multinomial logistic regression tests after adjustment for potential covariates. All statistical tests were two-sided with *p* < 0.05 taken to indicate statistical significance. Statistical analyses were performed using IBM SPSS Statistics (version 25; IBM, Chicago, IL).

## Results

### Recruitment and Treatment Flow

**Figure 1:**
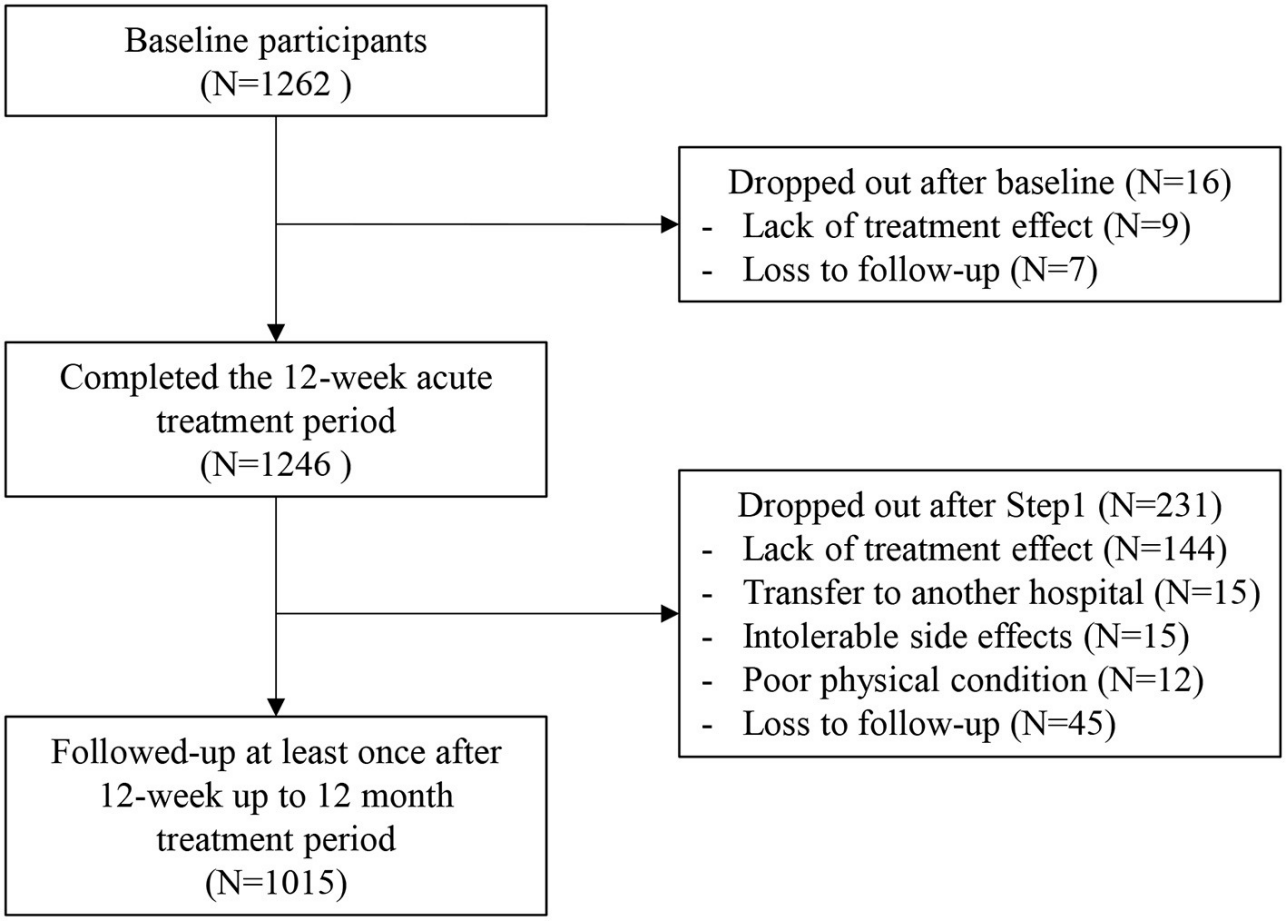
Participants flow chart.

Patient recruitment and flow are described in Figure 1. Of 1,262 patients evaluated at baseline, 1,246 (98.7%) were followed up at least once during the 12-week acute treatment phase, and comprised the sample analyzed for acute treatment outcomes. There were no significant differences in any baseline characteristic between the 1,246 patients included and 16 remaining patients (all *p* > 0.6).

After the acute treatment phase, 1,015 (81.5%) were followed up at least once during the treatment period, and comprised the sample analyzed for continuation treatment outcome. Attrition at 12 months was significantly associated with unemployed status and higher EQ-5D scores at baseline.

### Baseline Characteristics by Exposures

Characteristics were compared by presence of any childhood abuse in Table 1, which was significantly associated with younger age, male sex, higher education, unmarried status, no religion, and low monthly income. With regard to depression characteristics, the presence of childhood abuse was significantly associated with atypical features, early onset, longer duration of illness, greater number of depressive episodes, longer duration of present episode, family history of depression, and concurrent physical disorders. In addition, the presence of childhood abuse was significantly associated with higher baseline scores on HADS-A, LES, PSS, and AUDIT, and lower scores on both CDRS and MSPSS. There were significant group-related differences in treatment steps after 12 weeks. Characteristics were compared again according to the social support level using MSPSS (Supplementary Table S1). Lower social support was significantly associated with unmarried status, no religion, unemployed status, diagnosis of MDD, earlier age at onset, longer duration of illness, greater number of depressive episodes, longer duration of present episode, higher scores on HADS-D, HADS-A, and PSS, and lower scores on both SOFAS and CDRS. Considering these associations and collinearity between the variables, the following covariates were selected for further adjusted analyses: age, sex, atypical features, number of depressive episodes, duration of present episode, family history of depression, HADS-A, LES, and AUDIT scores, and treatment step.

**Table 1:** Baseline characteristics according to the presence of childhood abuse.

	**Absent (*N =* 1,063)**	**Present (*N =* 183)**	**Statistical coefficients**	***P*-value**
**Socio-demographic characteristics**				
Age, mean (SD) years	58.7 (13.8)	45.6 (17.6)	t = +9.583	<0.001
Sex, *N* (%) female	748 (70.4)	114 (62.3)	χ^2^ = 4.771	0.031
Education, mean (SD) years	8.9 (4.8)	10.8 (4.5)	t = −5.006	<0.001
Marital status, *N* (%) unmarried	297 (27.9)	86 (47.0)	χ^2^ = 26.626	<0.001
Living alone, *N* (%)	162 (15.2)	25 (13.7)	χ^2^ = 0.305	0.654
Religious affiliation, *N* (%) no	448 (42.1)	97 (53.0)	χ^2^ = 7.483	0.008
Unemployed status, *N* (%)	302 (28.4)	62 (33.9)	χ^2^ = 2.259	0.135
Monthly income, *N* (%) <2,000 USD	650 (61.1)	90 (49.2)	χ^2^ = 9.271	0.003
**Clinical characteristics**				
Major depressive disorder, *N* (%)	902 (84.9)	161 (88.0)	χ^2^ = 1.216	0.309
Melancholic feature, *N* (%)	168 (15.8)	18 (9.7)	χ^2^ = 4.379	0.043
Atypical feature, *N* (%)	53 (5.0)	29 (15.8)	χ^2^ = 29.956	<0.001
Age at onset, mean (SD) years	54.1 (15.6)	39.1 (17.9)	t = +10.617	<0.001
Duration of illness, mean (SD) years	4.6 (8.6)	6.5 (9.7)	t = −2.664	0.008
Number of depressive episodes, mean (SD)	1.0 (1.4)	1.7 (1.8)	t = −4.797	<0.001
Duration of present episode, mean (SD) months	6.9 (9.7)	10.2 (13.6)	t = −3.166	0.002
Family history of depression, *N* (%)	147 (13.8)	36 (19.7)	χ^2^ = 4.254	0.042
Number of physical disorders, mean (SD)	1.7 (1.3)	1.2 (1.3)	t = +4.188	<0.001
**Assessment scales, mean (SD) scores**				
Hospital anxiety & depression scale-depression subscale	13.6 (3.9)	13.6 (4.1)	t = +0.175	0.861
Hospital anxiety & depression scale-anxiety subscale	11.6 (4.1)	12.4 (4.0)	t = −2.561	0.011
EuroQol-5D	8.9 (1.5)	8.7 (1.5)	t = +1.238	0.216
Social and occupational functional assessment scale	56.0 (7.5)	55.1 (8.3)	t = +1.357	0.175
Life experiences survey	1.9 (1.5)	2.8 (2.6)	t = −4.145	<0.001
Perceived stress scale	26.8 (6.5)	28.4 (6.0)	t = −2.934	0.003
Connor-davidson resilience scale	43.6 (17.9)	39.5 (17.9)	t = +2.762	0.006
Multidimensional scale of perceived social support	39.8 (11.5)	35.5 (12.8)	t = +4.252	<0.001
Alcohol use disorders identification test	5.0 (8.5)	7.8 (10.5)	t = −3.399	0.001
**Treatment related characteristics**, ***N*** **(%)**			
Treatment step after 12 week				
Step 1	467 (43.9)	67 (36.6)	χ^2^ = 8.258	0.041
Step 2	352 (33.1)	60 (32.8)		
Step 3	188 (17.7)	38 (20.8)		
Step 4	56 (5.3)	18 (9.8)		

### Individual Effects of Childhood Abuse and Social Support Levels on Remission Status

Remission up to 12 weeks and up to 12 months were observed in 43.3 and 70.4% of cases, respectively. The individual associations of history of childhood abuse and social support group with the 12-week and 12-month remission status are shown in Table 2. All types of childhood abuse showed no association with acute 12-week remission in both unadjusted and adjusted analyses. However, all types of childhood abuse showed associations with long-term 12-month remission before and after adjustment. Higher social support was significantly associated with acute 12-week remission in unadjusted analyses, but this association lost significance after adjustment. There were no significant associations between social support and long-term 12-month remission in both unadjusted and adjusted analyses.

**Table 2:** Individual associations of reported childhood abuse and social support levels with remissions on 12-week and 12-month.

**Abuse type**	**Group**	**12-week remission**				**12-month remission**			
		**N**	**No. (%) remission**	**Unadjusted OR (95% CI)**	**Adjusted OR (95% CI)**	**N**	**No. (%) remission**	**Unadjusted OR (95% CI)**	**Adjusted OR (95% CI)**
Any abuse	Present	183	68 (37.2)	1.00	1.00	146	87 (59.6)	1.00	1.00
	Absent	1,063	472 (44.4)	1.35 (0.98–1.87)	1.08 (0.75–1.54)	869	628 (72.3)	1.77 (1.23–2.54)^†^	1.56 (1.04–2.33)^*^
Emotional abuse	Present	128	46 (35.9)	1.00	1.00	105	62 (59.0)	1.00	1.00
	Absent	1,118	494 (44.2)	1.41 (0.97–2.06)	1.16 (0.77–1.76)	910	653 (71.8)	1.76 (1.16–2.67)^†^	1.60 (1.01–2.54)^*^
Physical abuse	Present	122	47 (38.5)	1.00	1.00	99	55 (55.6)	1.00	1.00
	Absent	1124	493 (43.9)	1.25 (0.85–1.83)	1.02 (0.67–1.55)	916	660 (72.1)	2.06 (1.35–3.15)^†^	1.84 (1.16–2.93)^*^
Sexual abuse	Present	37	12 (32.4)	1.00	1.00	30	10 (33.3)	1.00	1.00
	Absent	1209	528 (43.7)	1.62 (0.80–3.25)	1.04 (0.50–2.18)	985	705 (71.6)	5.04 (2.33–10.89)^‡^	4.28 (1.89–9.73)^†^
Social support	Lower	659	2 67 (40.5)	1.00	1.00	535	374 (69.9)	1.00	1.00
	Higher	587	273 (46.5)	1.28 (1.02–1.60)^*^	1.21 (0.95–1.52)	480	341 (71.0)	1.06 (0.81–1.38)	0.99 (0.75–1.32)

*Adjusted for age, sex, atypical feature, number of depressive episodes, duration of present episode, family history of depression, scores on hospital anxiety & depression scale-anxiety subscale, life experiences survey, and alcohol use disorders identification test, and treatment step*.

* *P < 0.05;*

† *P < 0.01;*

‡ *P < 0.001*.

### Interactive Effects of Childhood Abuse and Social Support Levels on Remission Status

**Figure 2:**
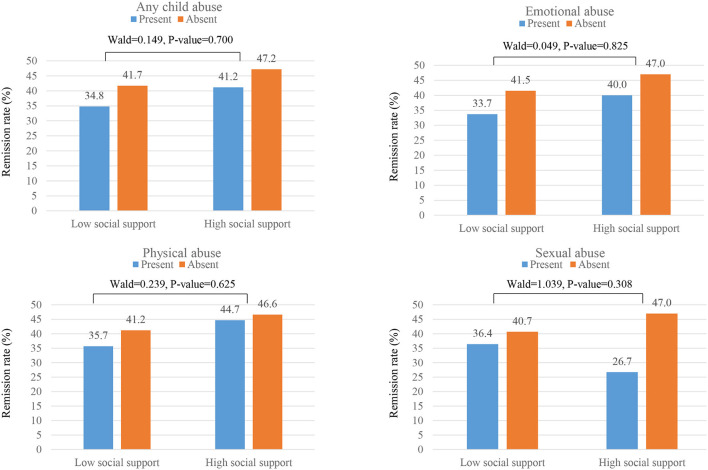
Interactive effects of childhood abuse and social support levels on 12-week remission rates. Interaction terms (Wald and P-value statistics) were calculated after adjustment for age, sex, atypical feature, number of depressive episodes, duration of present episode, family history of depression, scores on Hospital Anxiety & Depression Scale-anxiety subscale, Life experiences survey, and alcohol use disorders identification test, and treatment step.

**Figure 3:**
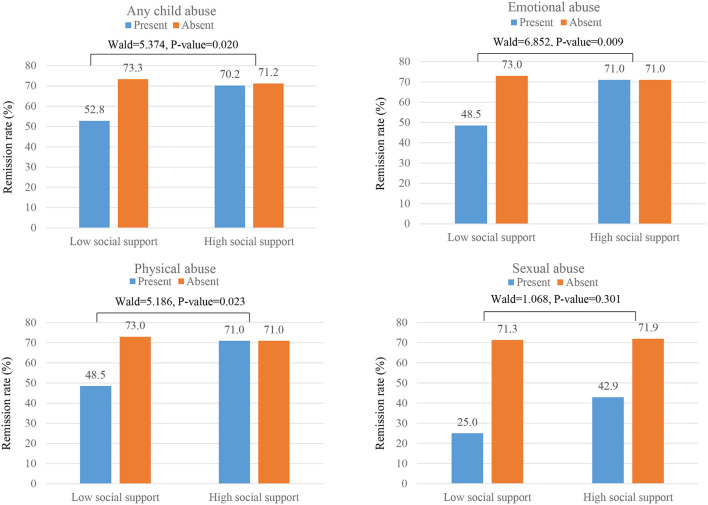
Interactive effects of childhood abuse and social support levels on 12-month remission rates. Interaction terms (Wald and P-value statistics) were calculated after adjustment for age, sex, atypical feature, number of depressive episodes, duration of present episode, family history of depression, scores on Hospital Anxiety & Depression Scale-anxiety subscale, Life experiences survey, and alcohol use disorders identification test, and treatment step.

The interactive effects of history of childhood abuse and social support levels on the 12-week remission status are shown in Figure 2. No significant interactive associations were found between each childhood abuse type and social support levels on the acute 12-week remission rate. Figure 3 shows the interactive effects of history of childhood abuse and social support levels on the 12-month remission status. Presence of both a history of child abuse and low level of social support was associated with the lowest long-term 12-month remission rates. Of these associations, interaction terms between any child abuse, emotional abuse, and physical abuse and baseline social support on remission rate were significant (*p* = 0.020, 0.009, and 0.023, respectively). However, no significant interactions were found with sexual abuse (*p* = 0.301).

## Discussion

The principal findings of this study were that childhood abuse before the age of 16 is associated with poorer 12-month long-term pharmacological treatment outcomes in patients with depressive disorders, but not in the 12-week acute treatment phase in adulthood. Furthermore, in analyzing the effects of the interaction of each type of childhood abuse and the level of social support on remission, we found interactive effects of history of childhood abuse and social support level on the long-term 12-month remission status. However, no interaction effect was found with sexual abuse.

This study included a sample of outpatients with depressive disorder from a naturalistic prospective pharmacological treatment protocol. Our findings were broadly inconsistent with previous studies reporting the association between childhood adversity and lack of remission in short-term (12 weeks) treatment (6). However, two studies of 12-month treatment outcomes showed that childhood abuse was associated with non-remission after a medication trial (49, 50) which was consistent with our 12-month results. As the present study is the first long-term study assessing both acute and late phases to report changes in the predictive value of child abuse for pharmacological treatment, our findings could provide a more integrated and expanded understanding in the context of previous research. Some studies exploring time to remission within several weeks indicated that there were differences in remission rates over time, and that childhood abuse was associated with longer time to remission across treatments (51, 52). From these findings, we can make assumptions about other mediators that impact the response to drug response in depressed patients with childhood abuse in the long-term rather than the short-term. A cross-sectional study to evaluate the 1-year outcome of depression during pharmacotherapy reported that coping with interpersonal stress rather than history of abuse was a significant long-term (1 year later) predictor of remission (49). Interpersonal events and their responses are factors that could change in the long-term along with responses to medications, and we speculate that they may have influenced long-term remission rather than acute remission *via* interaction with child abuse.

This speculation is an observation in the present study, which showed there was a significant interaction between perceived social support and childhood abuse and effects on remission of depression. With regard to treatment outcome, this is the first study to identify social support as a mediator of the association between childhood abuse and pharmacological outcome in the long-term. There are several possible explanations for the significant interactive effects of social support during drug therapy. First, it is possible that social support serves as an active coping strategy during pharmacological treatment (53). Perceived social support in contrast to received social support, defined as the actual provision of support, is the cognitive evaluation of being connected to others and knowing that support is available (54). Individual differences in cognitive processing of the abusive experience rather than the abuse itself may predict later psychological adjustment (54). This perceived social support can also be viewed as a coping strategy involving the effective use of a social support system, such as sharing feelings with family and friends and of talking to others about one's problems (55). The coping strategy regarding effective use of a social support system would involve sufficient communication between patients and healthcare providers and actively seeking change during drug therapy, and may be specifically associated with positive treatment outcome. Second, social support may confer resilience to chronic stress, such as child abuse, by modifying the physiological status, which may be associated with antidepressant treatment response. There is an emerging literature linking social support and neurobiological pathways, including cardiovascular, neuroendocrine, and immune systems (20). In particular, evidence for a gene–environment interaction involving social support suggested that social support may confer resilience to stress by moderating serotonin transporter (5-HTT) gene expression in maltreated children (56). Childhood abuse may increase the risk of depressive disorder through alteration of the HPA axis and CRF circuits in the brain (57). An animal study suggested that early life adversity and serotonin transporter gene variation interacted at the level of the adrenal gland to affect the adult HPA axis (58). Our findings suggest that social support may modify serotonin function, which enhances antidepressant response by affecting the regulation of HPA dysfunction due to early childhood adversity. Based on these results, we assume that social support during drug therapy modulates the risk of a history of child abuse by affecting coping strategies or neurobiological changes in the long-term rather than acute phase. These coping modalities and physiological changes *via* social support could be expected over a long-term of several months rather than short-term.

However, we found no interactive effects of sexual abuse and social support level on the long-term 12-month remission status. Childhood abuse may be one decisive source of heterogeneity that may also depend on trauma type (59). Some studies have suggested that specific types of abuse rather than a general history of trauma moderate pharmacological treatment response (60). Specifically, a previous study suggested that the buffering effect of social support on depression appeared to be diminished with increasing severity or complexity of maltreatment history (61). Child sexual abuse often co-occurs within the context of other types of family dysfunction, social deprivation, and physical and emotional abuse (62, 63). Considering the interaction between these additional stressors, the apparent association between child sexual abuse and adult depression is complex (63, 64). The findings of the present study suggest that the experience of sexual abuse may overpower the buffering effect of social support typically perceived, even during pharmacotherapy. Several studies have suggested that neurobiological consequences in adults who have experienced child sexual abuse are distinct from those of other types of abuse. A study of the HPA axis indicated that only sexual abuse during childhood, but not other types of child abuse, was correlated with cortisol dysfunction in pharmacological challenge tests in adults with posttraumatic stress disorder (65). Adults who had experienced childhood sexual abuse were shown to have cortical thinning, especially in regions of the primary somatosensory cortex, whereas adults who had experienced emotional abuse showed cortical thinning in regions of self-evaluation (66). These specific neurobiological consequences of childhood sexual abuse may have influenced the pharmacological response in a manner distinct from other types of child abuse by interacting with social support. Our findings suggest that appropriate social support interventions during medication for depressed patients with a history of sexual abuse cannot be expected to have a positive impact on the pharmacological outcomes. On the other hand, the findings of the present study bolster the idea that specific types of social support could exhibit effects on depression according to the type of child abuse. Previous research showed that actual parental support, but not cognitive appraisals or coping strategies, was predictive of resilience in victims of sexual abuse (67). That is, our findings suggest that perceived social support may not be beneficial in relation to the type of sexual abuse during medication for depression. Instead, patients with a history of sexual abuse may have better treatment outcomes by the provision of actual social support rather than facilitating cognitive appraisals about subjective social support. Therefore, research on whether such actual social support may have an impact on remission in depressed patients with a history of sexual abuse is required. In addition, special attention has been paid to ensuring the identification and provision of different types and sources of social support.

In addition, our findings agree with those of previous studies indicating that childhood abuse is associated with a pernicious course of depression, including early onset and greater number and persistence of depressive episodes (68, 69). In this study, no individual associations of social support level with the 12-week and 12-month remission status were found after adjusting for covariates. There are conflicting reports in the literature on the influence of social support on various health outcomes. Some researchers suggested that social support has a direct protective effect against depression (70), whereas others suggested that social support indirectly influences depression outcome *via* coping, communication, and self-esteem (71, 72). Our findings support the indirect effects of social support on treatment outcome in depressive patients. Future research should examine further the mechanisms affecting treatment outcomes during pharmacotherapy through social support and its relationship to childhood abuse.

The strengths of this study were that the sample size was larger and the follow-up period was longer than previous studies on this issue, and a range of covariates were considered in the analysis. However, this study had several limitations. First, as depressed patients in this study reported childhood abuse retrospectively, this information was vulnerable to the effects of recall and mood. In addition, the relatively small number of subjects with a history of sexual abuse compared to other types of childhood abuse may have underpowered the significant interactions with social support on remission status. However, several recent studies have found retrospective reports to be consistent with prospective designs (73, 74), and depressed individuals' reports of childhood abuse are stable despite changes in clinical state (75, 76). Second, follow-up rates for long-term treatment were relatively low compared with those for the acute treatment phase. Previous studies suggested that subjects with a history of childhood adversity are also more likely to drop out of treatment (77). Therefore, it is necessary to investigate the differences between types of child abuse among individuals who dropped out in the early phase and those of participants who remained until long-term follow-up. Third, as this study had a naturalistic design, treatment was decided by patient preference under physician's guidance rather than by a determined protocol; therefore, interphysician variability may have influenced the outcomes. Fourth, we did not assess the specific period associated with childhood abuse. Some studies suggested that specific periods (e.g., pre-pubertal age) of sensitivity to child abuse distinctly moderate the later response to pharmacotherapy (78). More work in the timing of childhood abuse and associated treatment outcomes is warranted.

In conclusion, we found that a history of child abuse predicted poorer pharmacological treatment response in depressed patients in the long-term. This study also indicated interactive effects of a history of childhood abuse and social support level on the long-term 12-month remission status. Notably, the level of social support showed no interactive effect with sexual abuse on the long-term treatment response to antidepressant medication. Our findings highlight the importance of early preventive and therapeutic interventions, and of obtaining information about childhood abuse in routine clinical assessments of adult patients with depression, to identify individuals at high risk of poor response to treatment with antidepressant medications.

## Data Availability Statement

The original contributions presented in the study are included in the article/Supplementary Material, further inquiries can be directed to the corresponding author.

## Ethics Statement

The studies involving human participants were reviewed and approved by Ethics Commission of the Chonnam National University Hospital Institutional Review Board (CNUH 2012-014). Written informed consent to participate in this study was provided by the participants' legal guardian/next of kin.

## Author Contributions

J-MK and RS were involved in the conception and design of the study and responsible for the acquisition of data. J-MK, RS, and J-YL were involved in the analysis and interpretation of data and performed the statistical analysis. J-MK and J-YL drafted the manuscript. H-JK, S-WK, and I-SS revised the manuscript critically for important intellectual content. H-JK, J-WK, and MJ contributed to administrative, technical, or material support. S-WK and I-SS supervised the study. All authors read and approved the final paper.

## Funding

The study was funded by a grant of National Research Foundation of Korea Grant [NRF-2019M3C7A1031345] to J-MK. RS is part-funded by the National Institute for Health Research (NIHR) Biomedical Research Centre at South London and Maudsley NHS Foundation Trust and King's College London, and from the National Institute for Health Research (NIHR) Applied Research Collaboration South London (NIHR ARC South London) at King's College Hospital NHS Foundation Trust. RS is also a National Institute for Health Research (NIHR) Senior Investigator.

## Conflict of Interest

The authors declare that the research was conducted in the absence of any commercial or financial relationships that could be construed as a potential conflict of interest.

## Publisher's Note

All claims expressed in this article are solely those of the authors and do not necessarily represent those of their affiliated organizations, or those of the publisher, the editors and the reviewers. Any product that may be evaluated in this article, or claim that may be made by its manufacturer, is not guaranteed or endorsed by the publisher.
