# Supplementary material for: Childhood Abuse, Social Support, and Long-Term Pharmacological Treatment Outcomes in Patients With Depressive Disorders
Source: Front Psychiatry. 2022 Feb 2;13:803639. doi: 10.3389/fpsyt.2022.803639 (PMC8847738; doi:10.3389/fpsyt.2022.803639)
Supplement: Supplementary file 1 [file Table_1.DOCX]

| **Table S1. Baseline characteristics according to the level of social support** | | | | |
| --- | --- | --- | --- | --- |
|  | Lower SS  (N=659) | Higher SS  (N=587) | Statistical  coefficients | P-value |
| **Socio-demographic characteristics** |  |  |  |  |
| Age, mean (SD) years | 56.2 (15.8) | 57.4 (14.4) | t=-1.508 | 0.132 |
| Sex, N (%) female | 455 (69.0) | 407 (69.3) | χ^2^=0.012 | 0.911 |
| Education, mean (SD) years | 9.0 (4.7) | 9.3 (4.9) | t=-1.305 | 0.192 |
| Marital status, N (%) unmarried | 228 (34.6) | 155 (26.4) | χ^2^=9.787 | 0.002 |
| Living alone, N (%) | 107 (16.2) | 80 (13.6) | χ^2^=1.656 | 0.198 |
| Religious affiliation, N (%) no | 318 (48.3) | 227 (38.7) | χ^2^=11.588 | 0.001 |
| Unemployed status, N (%) | 217 (32.9) | 147 (25.0) | χ^2^=9.337 | 0.002 |
| Monthly income, N (%) <2,000 USD | 403 (61.2) | 337 (57.4) | χ^2^=1.803 | 0.179 |
| **Clinical characteristics** |  |  |  |  |
| Major depressive disorder, N (%) | 580 (88.0) | 483 (82.3) | χ^2^=8.133 | 0.004 |
| Melancholic feature, N (%) | 109 (16.5) | 77 (13.1) | χ^2^=2.864 | 0.091 |
| Atypical feature, N (%) | 40 (6.1) | 42 (7.2) | χ^2^=0.595 | 0.441 |
| Age at onset, mean (SD) years | 50.7 (17.5) | 53.1 (16.0) | t=-2.541 | 0.011 |
| Duration of illness, mean (SD) years | 5.4 (9.2) | 4.3 (8.2) | t=+2.271 | 0.023 |
| Number of depressive episodes, mean (SD) | 1.2 (1.5) | 1.0 (1.5) | t=+2.007 | 0.045 |
| Duration of present episode, mean (SD) months | 8.3 (11.8) | 6.4 (8.5) | t=+3.137 | 0.002 |
| Family history of depression, N (%) | 93 (14.1) | 90 (15.3) | χ^2^=0.369 | 0.544 |
| Number of physical disorders, mean (SD) | 1.6 (1.3) | 1.6 (1.2) | t=+0.216 | 0.829 |
| **Assessment scales**, **mean (SD) scores** |  |  |  |  |
| Hospital Anxiety & Depression Scale-depression subscale | 14.2 (3.8) | 12.9 (4.0) | t=+6.026 | <0.001 |
| Hospital Anxiety & Depression Scale-anxiety subscale | 12.1 (4.0) | 11.4 (4.1) | t=+2.928 | 0.003 |
| EuroQol-5D | 8.8 (1.5) | 8.9 (1.5) | t=-1.433 | 0.152 |
| Social and Occupational Functional Assessment Scale | 55.4 (7.7) | 56.4 (7.5) | t=-2.407 | 0.016 |
| Life Experiences Survey | 2.1 (1.9) | 2.0 (1.5) | t=+0.475 | 0.635 |
| Perceived Stress Scale | 27.6 (6.3) | 26.5 (6.6) | t=+2.994 | 0.003 |
| Connor-Davidson Resilience Scale | 37.8 (16.9) | 48.8 (17.3) | t=-11.334 | <0.001 |
| Alcohol Use Disorders Identification Test | 5.4 (8.9) | 5.4 (8.9) | t=-0.092 | 0.927 |
| **Treatment related characteristics, N (%)** | |  |  |  |
| Treatment step after 12 week |  |  |  |  |
| Step 1 | 277 (42.0) | 257 (43.8) | χ^2^=0.413 | 0.938 |
| Step 2 | 220 (33.4) | 192 (32.7) |  |  |
| Step 3 | 122 (18.5) | 104 (17.7) |  |  |
| Step 4 | 40 (6.1) | 34 (5.8) |  |  |
